# eRF1 mediates codon usage effects on mRNA translation efficiency through premature termination at rare codons

**DOI:** 10.1093/nar/gkz710

**Published:** 2019-08-14

**Authors:** Qian Yang, Chien-Hung Yu, Fangzhou Zhao, Yunkun Dang, Cheng Wu, Pancheng Xie, Matthew S Sachs, Yi Liu

**Affiliations:** 1 Department of Physiology, The University of Texas Southwestern Medical Center, 5323 Harry Hines Boulevard, Dallas, TX 75390, USA; 2 Department of Biochemistry and Molecular Biology, National Cheng Kung University, Tainan 701, Taiwan; 3 State Key Laboratory for Conservation and Utilization of Bio-Resources and Center for Life Science, School of Life Sciences, Yunnan University, Kunming, Yunnan 650500, China; 4 Department of Biology, Texas A&M University, College Station, TX 77843-3258, USA; 5 Jiangsu Key Laboratory of Neuropsychiatric Diseases and Cambridge-Suda Genomic Resource Center, Soochow University, 199 Ren'ai Road, Suzhou, Jiangsu 215123, China

## Abstract

Codon usage bias is a universal feature of eukaryotic and prokaryotic genomes and plays an important role in regulating gene expression levels. A major role of codon usage is thought to regulate protein expression levels by affecting mRNA translation efficiency, but the underlying mechanism is unclear. By analyzing ribosome profiling results, here we showed that codon usage regulates translation elongation rate and that rare codons are decoded more slowly than common codons in all codon families in *Neurospora*. Rare codons resulted in ribosome stalling in manners both dependent and independent of protein sequence context and caused premature translation termination. This mechanism was shown to be conserved in *Drosophila* cells. In both *Neurospora* and *Drosophila* cells, codon usage plays an important role in regulating mRNA translation efficiency. We found that the rare codon-dependent premature termination is mediated by the translation termination factor eRF1, which recognizes ribosomes stalled on rare sense codons. Silencing of eRF1 expression resulted in codon usage-dependent changes in protein expression. Together, these results establish a mechanism for how codon usage regulates mRNA translation efficiency.

## INTRODUCTION

Most amino acids are encoded by two to six synonymous codons. Preference for certain synonymous codons, a phenomenon called codon usage bias, is a universal feature of all genomes (1–4). Codon usage bias is an important determinant of gene expression levels in both eukaryotes and prokaryotes (5–8). Selection for efficient translation is thought to be the major cause of codon usage bias (4,9–11); however, the molecular mechanism that mediates the codon usage influence on mRNA translation efficiency is unclear.

Codon optimization has long been used to enhance heterologous gene expression, and highly expressed proteins are mostly encoded by genes with strong codon biases for optimal codons. In addition, genome-wide correlations between protein levels and codon usage have been observed (12,13). Although codon optimization has been shown to alter polysome profiles of some mRNAs (8,14), which can be used as an indication of translation efficiency, the interpretation is complicated by the fact that codon usage also directly influences ribosome decoding rate (15–17). Furthermore, several recent studies suggest that translation efficiency is mainly determined by the efficiency of translation initiation, a process that is mostly determined by RNA structure but not codon usage near the translation start site (18–21). Adding to the complexity, codon usage has been shown to play an important role in determining mRNA levels by regulating transcription and mRNA stability (12,14,22–26). Furthermore, codon usage can also influence translation fidelity, splicing, and polyadenylation (4,23,27,28).

By using cell-free translation systems and ribosome profiling, we previously demonstrated that codon usage plays an important role in determining the translation elongation rate in both fungal and *Drosophila* cells (15,29). This conclusion was further confirmed by ribosome profiling and super-resolution microscopy studies (16,17,30,31). Codon usage regulates co-translational protein folding by affecting translation elongation rates in both prokaryotic and eukaryotic organisms (6,14,15,29,32–39). The effect of codon usage on elongation is likely caused by biases in tRNA expression profiles (40,41): It takes longer for rare codons to be recognized by cognate tRNA species present at low concentrations.

The role of codon usage on elongation rate may influence protein production in two ways. First, mRNAs with more optimal codon usage are translated more rapidly than those with less optimal codon usage, resulting in more protein production. However, protein initiation is usually the rate-limiting step in protein synthesis (21), so faster elongation does not necessarily result in more protein production. Second, rare codons can cause ribosome stalling, which may lead to premature termination of translation. Consistent with the latter mechanism, some codons have been shown to result in ribosome stalling (42–45). In addition, in *Escherichia coli* and *Saccharomyces cerevisiae*, the rare arginine codon CGA causes ribosome stalling (46–48).

Permanently stalled ribosomes can be rescued and released from mRNA. In yeast, Dom34 and Hbs1, which are related to the eukaryotic termination factors eRF1 and eRF3, trigger both release of stalled ribosomes and mRNA degradation through the no-go decay and nonstop decay pathways (49–51). Dom34 and eRF1 are both structural mimics of tRNA. Although Dom34 is related to eRF1, it lacks the NIKS codon recognition motif, which is responsible for discriminating between sense and stop codons, and it lacks a complete M domain which is necessary for hydrolysis of peptidyl-tRNA (52,53). The Dom34/Hbs1 complex plays a general role in quality-control systems by dissociation of a stalled ribosome at the 3′ end of aberrant mRNA and was previously proposed to rescue ribosome stalled at the tandem arginine CGA codons in yeast (49,50,54). The CGA codon is a unique case in yeast due to the lack of tRNA^UCG^ and its role in ribosome stalling is not conserved in other organisms (55). In addition, there was currently no evidence suggesting a role for Dom34/Hbs1 complex in ribosome rescue for other rare codons.

eRF1 and eRF3 mediate translation termination at stop codons (56–58). eRF1 enters the ribosome A site and is responsible for stop codon recognition, and eRF3 stimulates this process in a GTP-dependent manner. After stop codon recognition, the conformation of eRF1 changes; this conformational change induces hydrolysis of peptidyl-tRNA, release of nascent peptide, and subsequent steps that enable recycling of the ribosome (59,60). The high stringency of stop codon recognition by eRF1 is achieved by combined interactions of multiple domains in eRF1 with 18S rRNA and ribosomal proteins. Several motifs located at the apex of the N domain in eRF1 are essential for specific stop codon recognition (61–63). Although eRF1 has been shown to recognize several sense codons that mimic stop codons, the efficiency is much lower than that of stop codon recognition (64–66). While the efficiency of eRF1 recognition of sense codons is lower, here we show that ribosome stalling at rare sense codons can result in premature termination of translation mediated by eRF1 but not Dom34. These results, which show that protein synthesis can be abrogated by premature termination at rare codons, establish a heretofore unappreciated mechanism for how codon usage can control translation efficiency.

## MATERIALS AND METHODS

### Codon manipulation and indices calculation

Vectors for expression of *Luc* mRNA in *Neurospora* and *Drosophila melanogaster* with different codon-optimized regions were from previous studies (15,29). The codons of firefly *Luc* were optimized or deoptimized based on the *Neurospora* codon usage frequency calculated from the annotated coding sequences published in the Broad Institute *Neurospora crassa* database (http://www.broadinstitute.org/annotation/genome/neurospora/MultiHome.html). The *D. melanogaster* codon usage frequency table was obtained from (http://www.kazusa.or.jp/codon/cgi-bin/showcodon.cgi?species=7227 ).

### *Neurospora crassa* strains and culture conditions

The *Neurospora* strains used in this study for cell-free lysate preparation were wild-type (WT) 74A-OR23–1VA (A). *dom34^KO^* was generated by replacing the *dom34* gene with the *hygromycin resistance* gene by homologous recombination (67). The *dim-5^KO^* (*bd, his-3*) strain was used as the host strain for all *his-3* targeting constructs to monitor eGFP-2A-Luc reporter expression *in vivo* (12). The eRF1-KD strain was generated in the 301–6 (*bd, his-3, a*) background as described previously (68). To induce eRF1 silencing, *Neurospora* mycelium was cultured in 0.01 M quinic acid with 1 × Vogel's, 0.1% glucose and 0.17% arginine overnight at 25°C.

### Plasmid construction

Codon optimization was performed as described previously (15,29). Expect for the first 10 amino acids, the full-length OPT-Luc and DeOPT-Luc constructs contain the most or the least preferred codons, respectively. For constructs used in *Neurospora* and *Drosophila* cell-free assays, the *Luc* sequence was cloned into pJI204 vector containing a T7 promoter and 30-nt poly-A sequence at BamHI/XbaI sites. Other *Luc*-based constructs were created by homologous recombination-based cloning (In-Fusion HD cloning kit, Clontech) using the WT-Luc, OPT-Luc, or DeOPT-Luc as parental templates.

The *Neurospora* eGFP-2A-Luc constructs for *in vivo* study were generated in pMF272 plasmids, which contain the *ccg-1* promoter and *his-3* targeting sequence by insertion at the AscI/XbaI. *Drosophila* S2 *in vivo* constructs were created on the backbone of pAc-STABLE1-neo (69). Different versions of *Luc* sequences were inserted into the pAc vector at NheI/XhoI sites.

### *In vitro* transcription

To prepare the templates for *in vitro* transcription, the plasmids were linearized by treatment with NheI followed by successive phenol-chloroform extraction and ethanol precipitation. The capped and poly-A tailed mRNA transcripts were then synthesized using HiScribe T7 Quick High Yield RNA Synthesis Kit (NEB) supplemented with 3′-O-Me-m7G(5′)ppp(5′)G anti-reverse cap structure analog (NEB) per manufacturer's instructions. The integrity and quantity of the synthesized transcripts were evaluated by denaturing agarose gel electrophoresis. The mRNA concentrations were determined using a Nanodrop spectrophotometer (ThermoFisher Scientific).

### Preparation of *Neurospora* cell-free translation extract and *in vitro* translation reactions

The preparation of WT translation extract was performed following a protocol previously described (70). *Neurospora* conidia were harvested and inoculated at a concentration of 1 × 10^7^ conidia/ml in 1 × Vogel's with 2% sucrose. A 1-L culture was incubated at 32°C with shaking at 200 rpm for 6.5 h. Geminating conidia were harvested by vacuum filtration and snap frozen in liquid nitrogen. The mycelial pads were homogenized using a mortar and pestle with gradual addition of one volume of Buffer A (30 mM HEPES-KOH, pH 7.6, 100 mM KOAc, 3 mM Mg(OAc)_2_, 2 mM DTT, 1 × protease inhibitor cocktail (A32963, ThermoFisher Scientific)). The lysate was centrifuged for 15 min at 30 000 × *g* at 4°C. The supernatant was carefully collected and centrifuged again. Small molecular weight molecules were removed using Zeba Desalt Spin Columns (Pierce). For preparation of extract from eRF1-KD strains, the mycelium mat was cultured in 0.01 M quinic acid with 1 × Vogel's, 0.1% glucose, and 0.17% arginine overnight at 28°C. The lysate was aliquoted, snap frozen in liquid nitrogen and stored at −80°C.

The *in vitro* translation was performed as previously described (15). A reaction mixture (5 μl of cell lysate with 1 μl of 10 × energy mix, 0.06 μl of 10 U creatine phosphate kinase, 0.35 μl of 2 M KOAc, 0.1 μl of 0.1 M Mg(OAc)_2_, 0.1 μl of 1 mM amino acids mix, 0.1 μl of RNase inhibitor (ThermoFisher Scientific) and 2.4 μl of RNase-free water) was prepared, and 60 ng of mRNA was added to achieve a total volume of 9.5 μl. [^35^S]Met (0.5 μl/reaction) was added to label all protein products. The reaction was incubated at 26°C for various time. For RNase A treatment, 1 μl of 10 mg/ml RNase A (ThermoFisher Scientific) was added to reaction and incubated for 15 min at 37°C. For puromycin treatment, 1 μl of 10 mg/ml puromycin (Sigma) was added and the reaction was incubated at 26°C for 15 min. For harringtonine treatment, 1 μl of 10 mg/ml harringtonine (Sigma) was added after 6 min, and the reaction was incubated at 26°C for an additional 9 min. To determine eRF1 function, 0.6 μg recombinant protein was added to 20 μl *in vitro* translation reaction (0.6 μM). The eRF1 concentration used in our assays is similar to previous studies (71,72). The reactions were stopped by placing on ice and were analyzed by electrophoresis on 10% NuPAGE gel (ThermoFisher Scientific).

### S2 cell culture and transfection

*Drosophila* S2 cells were cultured in Schneider's *Drosophila* Medium (Gibco) supplemented with 10% heat-inactivated fetal bovine serum (Gibco) and 1% penicillin-streptomycin (10 000 units penicillin and 10 mg streptomycin/ml, Sigma) at 27°C. For transfection, cells were plated at 5 × 10^6^ cells per well of a 12-well plate. For expression of reporter genes, 1 μg plasmid was transfected into each well using Lipofectamine 3000 (ThermoFisher Scientific) following the manufacturer's instructions. Cells were harvested after 48 h for further analysis.

### Preparation of S2 cell-free translation extract and *in vitro* translation

The preparation of S2 extract and *in vitro* translation were performed as previously described (29). After reaching confluence, S2 cells were harvested by centrifugation at 1000 × *g* for 4 min and washed with 1 × phosphate buffered saline three times. Cell pellets were resuspended in 2 × volumes of hypotonic buffer (10 mM HEPES-KOH pH 7.6, 10 mM potassium acetate, 0.5 mM Mg(OAc)_2_, 5 mM dithiothreitol) and incubated on ice for 40 min to 1 h. Cells were then homogenized by 20–30 strokes in a Dounce homogenizer placed on ice, and the final concentration of KOAc was adjusted to 50 mM. The cell extract was centrifuged at 16 000 × *g* for 10 min at 4°C. The supernatant was aliquoted, snap frozen in liquid nitrogen and stored at −80°C.

### Isolation of ribosome-associate nascent peptides

The *in vitro* translation reactions were terminated by adding cycloheximide to a final concentration of 0.5 mg/ml. For cell culture, S2 cells were treated with cycloheximide (0.01 mg/ml) for 10 min and harvested. S2 cells were lysed in hypotonic buffer and homogenized by a Dounce homogenizer. Translation product or cell lysate was carefully layered on top of a sucrose cushion (0.5 M sucrose, 25 mM HEPES, pH 7.5, 80 mM KOAc, 1 mM Mg(OAc)_2_) and then centrifuged in a TL100.3 rotor (Beckman Coulter) at 95 000 rpm for 15 min at 4°C. The ribosomal pellets are washed once with 25 mM HEPES (pH 7.5), 80 mM KOAc, and 1 mM Mg(OAc)_2_ and then resuspended in lysis buffer for further analysis.

### Protein expression and purification

The plasmid pQE2 (Qiagen) was used for recombinant protein expression. The *eRF1* open reading frame was amplified by polymerase chain reaction (PCR) amplified from *Neurospora* cDNA and inserted into pQE2 at the NheI/NcoI site. Site directed mutagenesis by PCR was used to generate mutant *eRF1*. The constructs were overexpressed in *E. coli* BL21. The recombinant protein was purified by affinity chromatography on nickel beads (Sigma) according to the manufacturer's protocol. The protein concentration was determined using a Nanodrop spectrophotometer.

### Protein analysis

*Neurospora* tissue harvest, protein extraction and western blot analysis were performed as previously described (12). S2 cells were lysed in 1 × Passive Lysis Buffer (Promega) according to the manufacturer's instruction. For western blot analyses, equal amounts of total protein were loaded in each lane. After electrophoresis, proteins were transferred onto PVDF membrane and western blot analysis was performed. Anti-Flag antibody (F3165, Sigma) was used to detect Flag-tagged proteins, anti-luciferase antibody (L2164, Sigma) was used to detect LUC and anti-Myc antibody (M4439, Sigma) was used to detect Myc-tagged proteins.

### RNA analysis

RNA was extracted with Trizol (Ambion) in accordance with the manufacturer's protocol. For qRT-PCR, the primer sequences used for amplification of *Luc* in *Neurospora* targeting the 5′UTR and the flag-tag region: 5′-ACCCCTCACATCAACCAAAGG-3′ (forward) and 5′-GCCGCCCTTGTCATCGTCATC-3′ (reverse). The *Neurospora* gene coding for β-tubulin was used as an internal control. The primer sequences for *β-tubulin* amplification were 5′-ATAACTTCGTCTTCGGCCAG-3′ (forward) and 5′-ACATCGAGAACCTGGTCAAC-3′ (reverse). The primer sequences used for amplification of *Luc* in *Drosophila* targeting the 5′UTR and the myc-tag region: 5′-TGATATCATCGATTTAAAGCAATGGAG-3′ (forward) and 5′-GTCGCCCAAGCTCTCCATTTCATT-3′ (reverse). The *Drosophila* gene coding for actin was used as an internal control. The primer sequences for *actin* amplification were 5′-GCACACCCACAAGCTTACACA-3′ (forward) and 5′-TTGCGCTTTGGGAAATATCTTC-3′ (reverse).

### Analysis of ribosome profiling results

The ribosome profiling and RNA-seq experiments were previously performed and cycloheximide was not added in cell culture and was only added in extraction buffer (15). The results were analyzed to determine relative codon decoding time (RCDT) of each codon. We only selected the mapped ribosome protected fragments (RPF) of 28–32 nt long, which accounted for 90–94% all reads over 25 nt. The ribosomal A sites where codon selection occurs were considered as nucleotide positions 16–18 and the 5′ most nucleotide of each sequenced reads was assumed to be position 1 as described previously (73). We then calculated the fraction of in-frame codons by comparing the read sequences with annotated *Neurospora* coding sequences. A vast majority (∼80%) of codons at positions 16–18 of sequenced RPFs are in-frame. All out-of-frame reads were excluded from the analyses. The trimmed alignment files were processed with bedtools to generate normalized bedgraph files containing the footprints of codons at ribosomal A site. To determine the mRNA level of each gene, we counted the short reads from RNA-seq data that had at least have one nucleotide overlapped with the open reading frame (ORF). The sum of the reads was normalized by the length of mRNA, and the library size was scaled to 1 million RPKM. The library size is based on the total reads mapped to identified transcripts and does not include rRNA or tRNA.

The RCDT value of each sense codon was derived as follows: For a gene with RPKM ≥ 1, if a specific codon has at least 1 RPF read covered and is not located within the first or last 20 amino acids from N or C terminus, respectively, its normalized RPF reading (i.e. RPM) was divided by the mRNA density (RPKM) determined by RNA-seq. We summed all quotients of this codon from the genome and divided by the total number of codons in the analysis, to determine the codon decoding time (CDT) for each sense codon. To calculate RCDT, the CDT of each codon was normalized to the highest occupied codon, which was CCA. The analyses of ribosomal profiling data were achieved through a collection of customized scripts written in Perl, which are available upon request.

## RESULTS

### The codon usage effect on ribosome occupancy *in vivo* revealed by ribosome profiling

**Figure 1.**
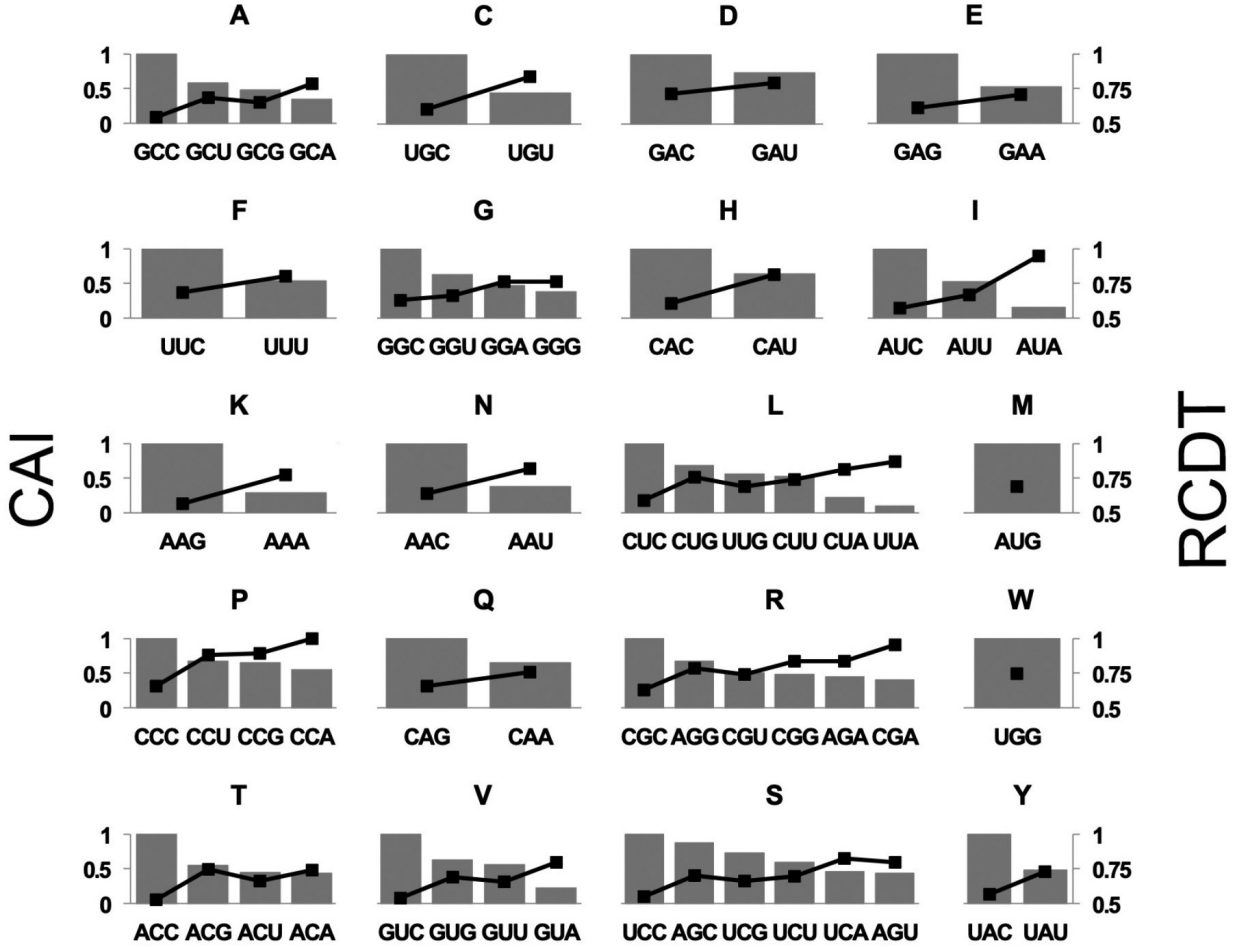
Relative codon decoding time negatively correlates with codon usage frequency in all codon families with more than two synonymous codons. Plotted are CAI (bars, left axis) and RCDT (black dots, right axis) for each codon family. Similar results were obtained from multiple independent replicate experiments.

We previously performed ribosome profiling on mRNAs with various patterns of codon usage at a high signal-to-noise ratio and demonstrated that codon usage plays an important role in regulating translation elongation rate in *Neurospora* (15). Here we analyzed the ribosome profiling results in *Neurospora* and compared the relative codon decoding time (RCDT) within each codon family. The RCDT value for each codon was determined by normalizing the quantity of ribosome protected fragments (RPF) to that of the most occupied codon (CCA, which encodes proline) and reflects the average relative dwell time of ribosome on the specific codon. For all 18 of the amino acid codon families with at least two synonymous codons, a negative correlation was observed between codon adaptation index (CAI) and RCDT (Figure 1). Importantly, the most frequently used codon in each family was always the one with the lowest RCDT value. Given that A-site occupancy assessed globally for individual codons by ribosome profiling should be generally indicative of the speed at which an individual codon is decoded, these results demonstrated that codon optimality affects translation elongation rate and that frequently used codons are generally decoded faster than non-preferred codons for all codon families in *Neurospora*.

### Non-optimal codons cause ribosome pausing during translation in both amino acid sequence context-independent and sequence context-dependent manners

**Figure 2.**
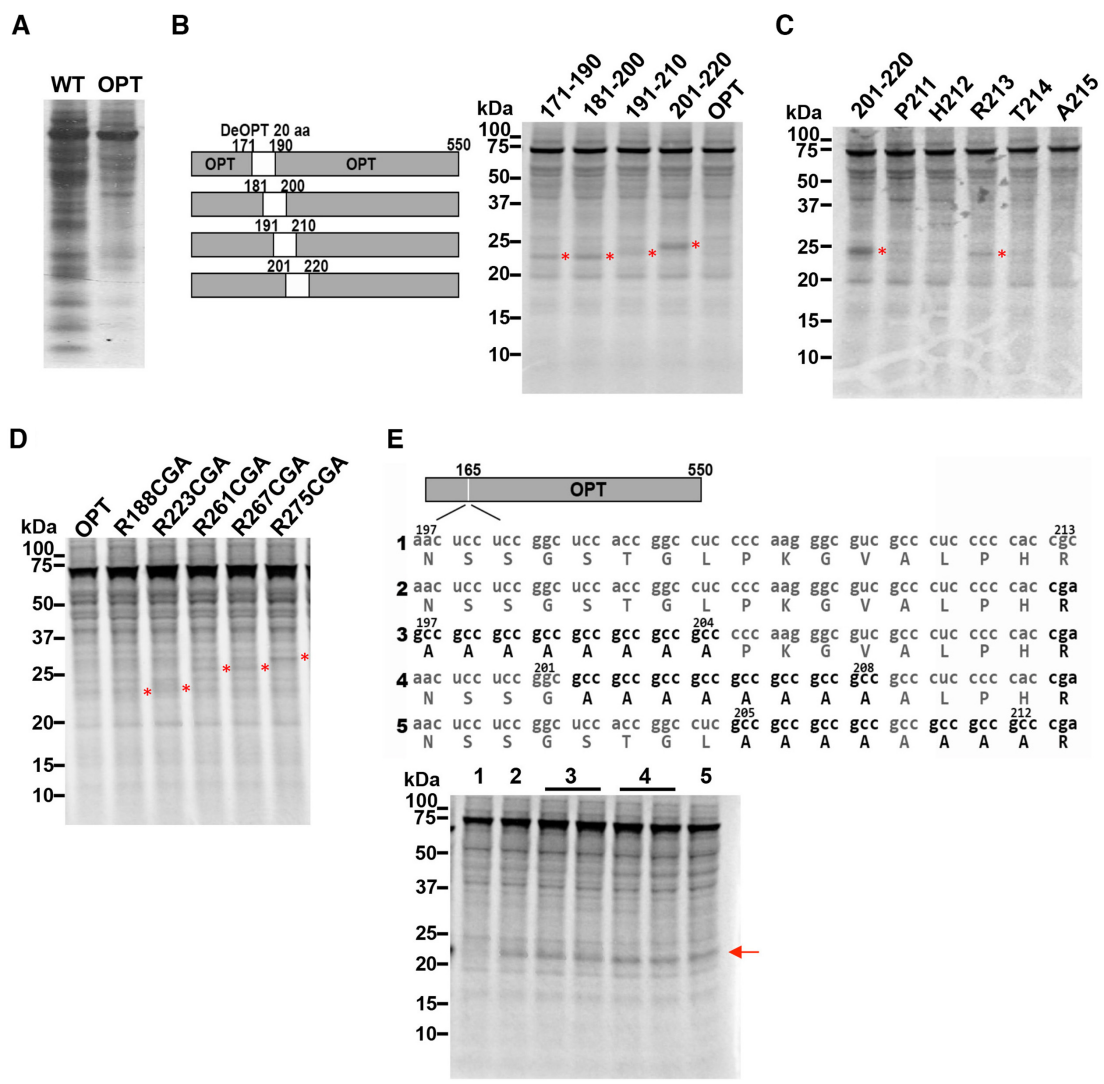
Rare codons cause ribosome stalling in an amino acid sequence-independent manner. (**A**) [^35^S]-Met-labeled total translation products analyzed by electrophoresis on 10% NuPAGE gel. Translation of WT and OPT *Luc* mRNAs was carried out for 12 min. (**B**) Left panel: Diagrams showing the 20-amino-acid scanning constructs. Codon-deoptimized (using the least used codons according to the *Neurospora* codon usage table) regions are indicated in white. Right panel: [^35^S]-Met-labeled total translation products after 12 min of translation analyzed by electrophoresis on 10% NuPAGE gel. The asterisks indicate the nascent peptide bands that appeared upon codon deoptimization. (**C**) [^35^S]-Met-labeled total translation products analyzed by electrophoresis on 10% NuPAGE gel. Lanes are labeled with the individual codon that was deoptimized in the OPT *Luc* mRNA background. The asterisks indicate the peptide bands that appeared upon introduction of rare codons. (**D**) [^35^S]-Met-labeled total translation products analyzed by electrophoresis on 10% NuPAGE gel. Lanes are labeled with the individual codon that was deoptimized in the OPT *Luc* background. (**E**) Top panel: Diagrams showing the sequences inserted at position 165 of the OPT *Luc* construct. Bottom panel: [^35^S]-Met-labeled total translation products analyzed by electrophoresis on 10% NuPAGE gel. Lane numbers correspond to numbers in the upper diagram. The arrow labels the band that appears due to insertion of the sequence with a CGA codon. Similar results were obtained from multiple independent replicate experiments.

To further determine the effect of codon usage on translation elongation, we examined the production of nascent peptides by *in vitro* translation assays using a *Neurospora* cell-free translation system that was previously shown to accurately reflect protein synthesis *in vivo* (15,74–78). Endogenous mRNAs were depleted from this system by nuclease digestion so that the synthesis of nascent polypeptides from exogenously supplied template mRNA can be labeled and visualized using [^35^S]-methionine. Two versions of firefly *luciferase* (*Luc*) mRNA were used as templates: WT (CAI of 0.65) and OPT. In OPT, all except the first 10 codons of the *Luc* open reading frame were changed to the most preferred codons in *N. crassa*. As shown in Figure 2A, the [^35^S]-methionine labeled protein products of the WT and OPT mRNAs after 12 min of translation exhibited markedly different profiles. In addition to the full-length LUC protein, translation of the WT *Luc* mRNA resulted in many non-full-length LUC species. For the OPT mRNA, however, the levels of these shorter peptides either decreased dramatically or disappeared. Comparison of the *in vitro* translation products at different time points after addition of CHX showed that intermediate peptides are not protein degradation products because both the full-length and intermediate protein bands were stable in the extracts during the time-frame of the reactions (Supplementary Figure S2A). These results suggest that non-optimal codons result in ribosome stalling or pausing that led to the various shorter than full-length protein products.

To further determine the role of rare codons in causing ribosome stalling or pausing, we converted codons in overlapping 20 amino acid windows from amino acid (aa) 171 through aa 220 in the OPT *Luc* mRNA to the least preferred codons in *Neurospora* and performed *in vitro* translation (Figure 2B, left). Deoptimization of codons in this region resulted in the accumulation of peptides with molecular weights corresponding to predicted locations of codon deoptimization (right panel of Figure 2B and Supplementary Figure S1A). To identify the codons most responsible for the ribosome stalling, we created mRNAs with single codon substitutions within the region from aa 211 through aa 215. We found that a single rare arginine codon CGA at R213 caused accumulation of the specific intermediate band (Figure 2C, lane 4). In addition, substitutions of arginine codons individually with the rare CGA codon at several upstream and downstream positions (R188, R223, R261 and R275) also caused accumulation of nascent peptides of the predicted sizes (Figure 2D and Supplementary Figure S1C). In addition, when we inserted the sequence from aa 197 through aa 213 of *Luc* containing R213 into a location upstream (aa 165) of *Luc* mRNA, CGA but not CGC at R213 resulted in the appearance of a nascent peptide band of the predicted size (Figure 2E and Supplementary Figure S1D). Furthermore, when the 16 amino acids upstream of R213 were divided into three parts and each was mutated to optimal alanine codons (GCC) to eliminate potential peptide interactions with ribosome exit tunnel, the accumulated nascent peptide was still observed. Together, these results demonstrate that the rare CGA codon alone is sufficient to cause significant ribosome stalling in a manner that is independent of both RNA sequence context and nascent polypeptide sequence context. The cognate tRNA (tRNA^ARG(UCG)^) for decoding CGA codon is rare in *Neurospora*: there are two copies of the gene encoding tRNA^ARG(UCG)^ (out of 36 tRNA^ARG^ genes) in the genome. In *S. cerevisiae*, CGA repeats were previously shown to inhibit gene expression due to the lack of a tRNA^ARG(UCG)^ gene to provide an optimal tRNA for decoding CGA; CGA can only be decoded inefficiently by tRNA^ARG(ICG)^ through A-I base pairing (46,47).

**Figure 3.**
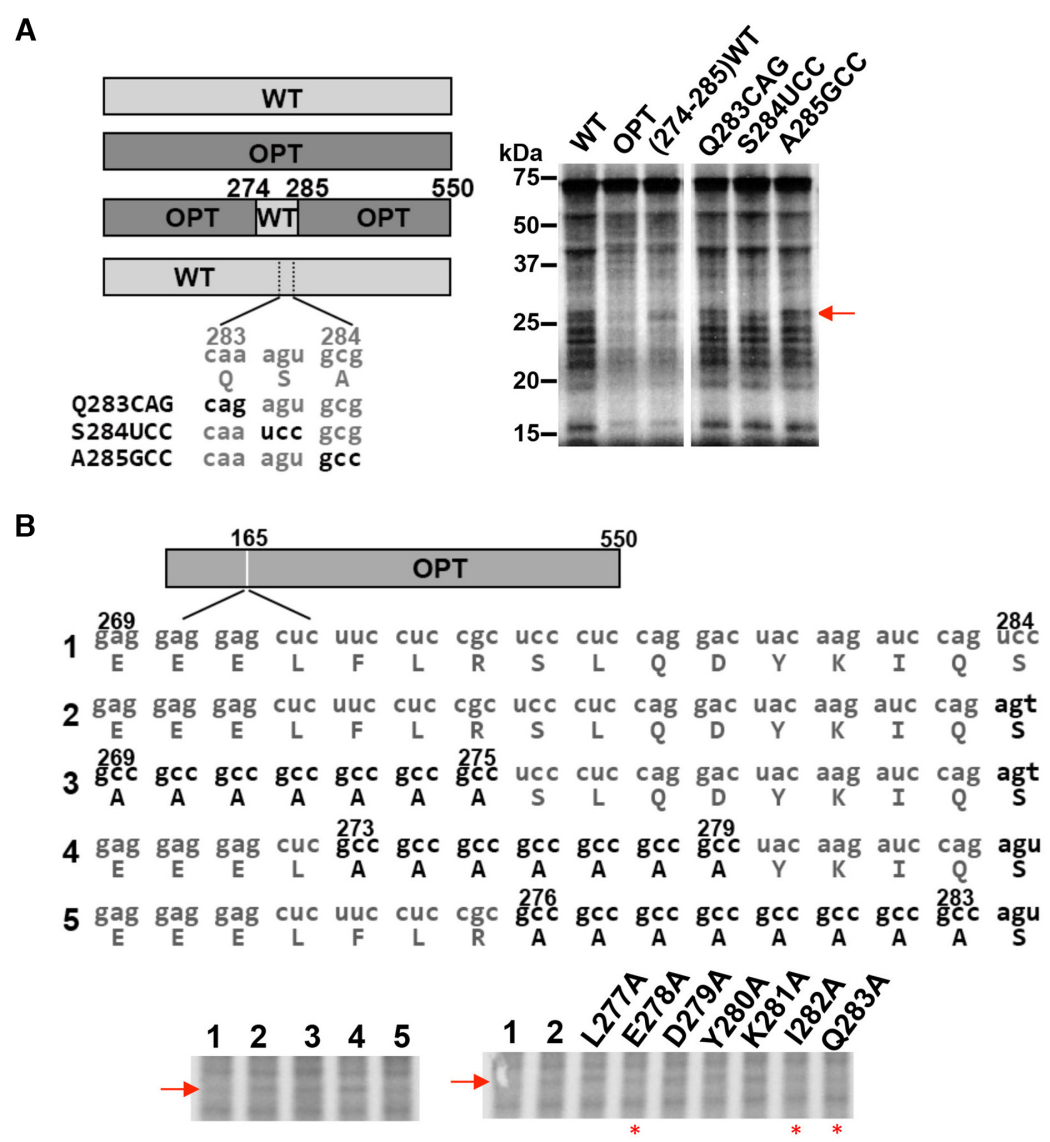
Rare codon causes ribosome stalling in a manner dependent on protein sequence context. (**A**) Left panel: Diagrams showing the rare codons introduced in different *Luc* constructs. Right panel: [^35^S]-Met-labeled total translation products of the indicated mRNAs. Red arrow labels nascent peptide produced due to the presence of a rare codon. (**B**) Top panel: Diagrams showing the sequences of constructs used to introduce alanine regions upstream of a rare codon. Bottom panel: [^35^S]-Met-labeled total translation products of the indicated mRNAs. Red arrow labels nascent peptide band caused by rare AGU codon. Red asterisks label the samples with reduced accumulation of the band. Similar results were obtained from at least one replicate experiment.

To identify additional codons responsible for ribosomal stalling, we replaced aa 274 through aa 285 in the OPT *Luc* mRNA with WT codons. The WT codons were responsible for the accumulation of doublet bands of nascent peptides at the predicted positions of rare codons (Figure 3A, compare lane 1 to lanes 2 and 3). Further mutation of some of the individual WT codons within this region to the common codon indicated that the rare serine AGU codon at 284 (lane 5) is required for the accumulation of the upper band of the doublet. To confirm this result, we insert five versions (Figure 3B, top) of the region from aa 269 to aa 284 at position 165 in the OPT *Luc* mRNA (Figure 3B, lower left lanes 1–5). For the second version, all codons in this region were optimal codons with S284 was encoded by the rare AGU codon. As predicted, the introduction of the S284 AGU codon resulted in the accumulation of a nascent peptide band at the expected size, confirming that the rare AGU codon is sufficient to cause ribosome stalling (Figure 3B, lower left panel).

To determine whether the effect of AGU is influenced by its upstream amino acid sequence, we mutated aa 269–275 (lane 3), aa 273–279 (lane 4) and aa 276–283 (lane 5) of OPT *Luc* RNA with the rare AGU codon at S284 separately to optimal GCC alanine codons. Mutation of aa 276–283 (lane 5) to alanines caused a marked reduction of the level of the accumulated nascent peptide product, whereas mutation of the other regions had little effect (Figure 3B lower left, and Supplementary Figure S1F). We then individually mutated each amino acid in the region from aa 276–283 to alanine (GCC) and found that the mutation of either I282 or Q283 reduced the accumulation of the band. Mutation of E278 also reduced the accumulation of the band (Figure 3B lower right panel, and Supplementary Figure S1G). Together, these results indicate that the upstream amino acid sequence also contributes to ribosome stalling at the rare AGU codon. This effect might be due to that the ability of the upstream amino acids to interact with the ribosome exit tunnel, which can affect translation elongation process (44,79).

### Non-optimal codons cause premature translation termination and reduce translation efficiency in *Neurospora*

The intermediate-length nascent peptides we observed could be peptidyl-tRNA intermediates associated with paused ribosomes that can continue translation when the empty A sites are recognized by tRNA or these peptides could be prematurely terminated translation products. To distinguish stalled and premature terminated polypeptides during translation, we established RNase A treatment assay and sucrose cushion assay by translating a stop-codon-less reporter mRNA that was known to produce stalled peptidyl-tRNAs on the poly(A) tail (Supplementary Figure S2B). On sodium dodecyl sulphate-polyacrylamide gel electrophoresis with neutral pH (NuPAGE) which can prevent alkaline hydrolysis of peptidyl-tRNA (54,80), the terminated nascent peptide should migrate at molecular weight ∼23 kDa, whereas the peptidyl-tRNA species should be ∼20 kDa larger. As shown in Supplementary Figure S2B, the majority of the translation products are ∼43 kDa which were converted to ∼23 kDa after RNase A treatment. In addition, after the translation products were subjected to 0.5 M sucrose cushion centrifugation, the untreated ∼43 kDa species resided in the pellet, confirming that they were the peptidyl-tRNA associated with ribosome. On the other hand, the RNase A treatment products mostly resides in the supernatant. This result demonstrates that RNase A and sucrose cushion assays could be used to distinguish stalled and premature terminated polypeptides.

**Figure 4.**
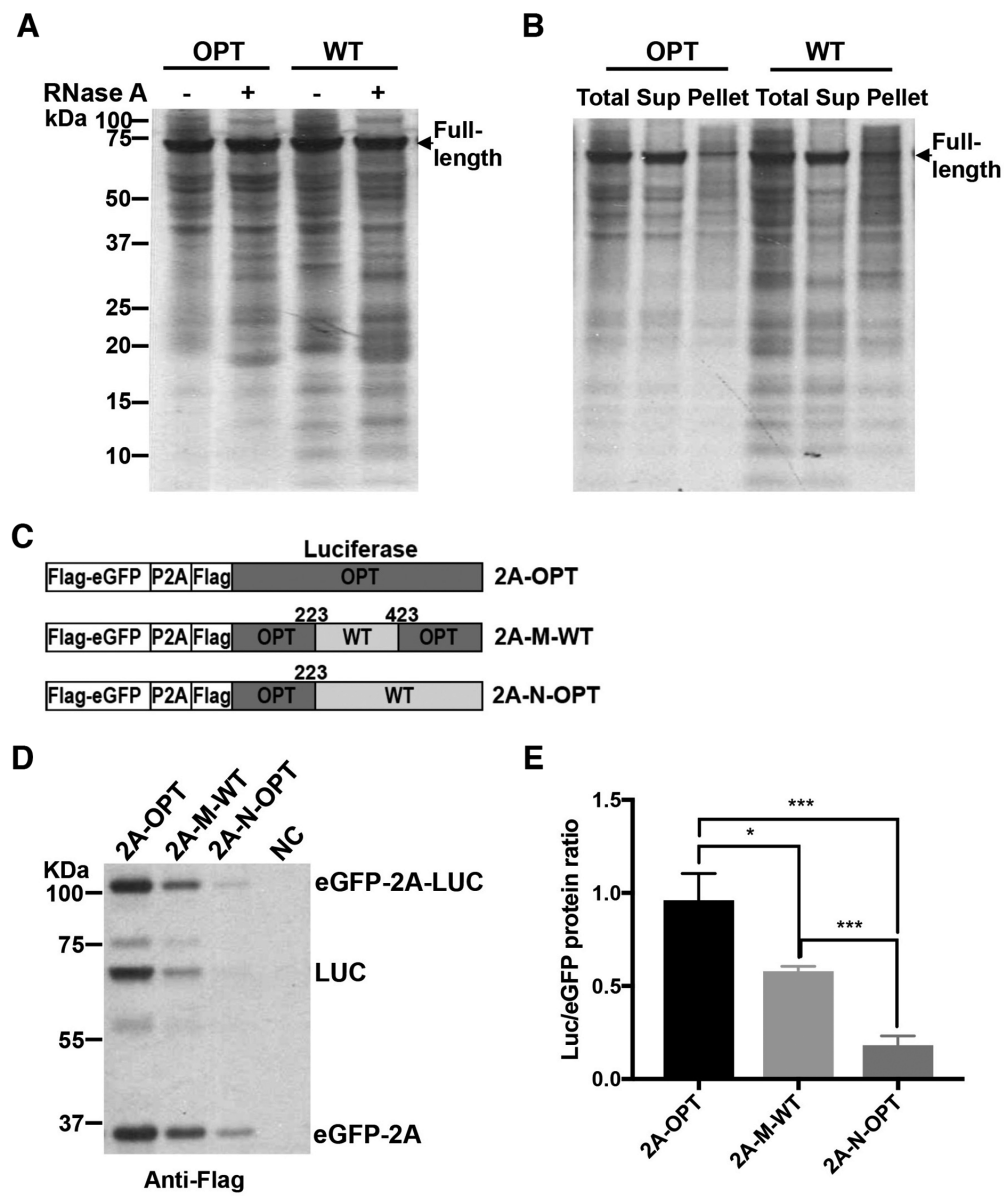
Rare codons cause premature termination and reduce translation efficiency. (**A**) [^35^S]-Met-labeled total translation products of WT and OPT *Luc* mRNA with and without RNase A treatment. After 10 min of translation, samples were treated with RNase A for 15 min at 37°C. (**B**) Pellet- and supernatant-associated [^35^S]-Met-labeled translation products of WT and OPT *Luc* mRNAs. After 10 min of translation, reactions were terminated by addition of cycloheximide (0.5 mg/ml final concentration). Ribosome-associated nascent peptides were separated by sucrose cushion centrifugation. Sup: supernatant. Similar results were obtained from multiple replicates for experiments described in (A) and (B). (**C**) Diagrams of eGFP-2A-Luc reporter constructs. (**D**) Representative western blot analysis of the eGFP-2A-Luc translation products. *Neurospora* strains carrying the indicated reporter construct were grown, and proteins were extracted for analysis. The positions of the eGFP-2A, Luc and full-length eGFP-2A-Luc proteins are labeled. NC: negative control, which was expression of empty vector. (**E**) Quantification of western blot analysis shown in panel (D) and two additional independent experiments. The ratio of LUC to eGFP is plotted. Data are means ± SD. *P < 0.05. ***P < 0.001.

To examine the *in vitro* translation products of the WT and OPT *Luc* mRNAs, we halted the *Luc* translation reactions at 12 min and treated the samples with RNase A, which releases nascent peptide chains from tRNAs. If peptides are linked to tRNA, RNase A treatment results in mobility shifts to lower molecular weight products on NuPAGE gel. On the other hand, nascent peptide chains that do not show mobility shifts should be premature termination products. Although a few nascent LUC peptide bands resulting from translation of WT *Luc* exhibited mobility shifts after RNase A treatment, the majority did not (Figure 4A), indicating that most of the observed shorter polypeptides were premature termination products. For the OPT *Luc* mRNA translated products, RNase A treatment led to the appearance of many lower molecular weight products, suggesting that many of these species are present as peptidyl-tRNAs and are thus translation intermediates.

To further confirm this conclusion, we analyzed the *Luc* translation products by centrifugation of translation reactions by sucrose cushion. As expected, we found the nascent intermediate peptide levels were higher from the WT *Luc* mRNA translation reaction in the supernatant than observed for the OPT *Luc* mRNA translation reaction (Figure 4B). There were also more nascent peptide bands in the WT pellet than the OPT pellet, suggesting that there were more stalled peptidyl-tRNAs in the WT. These results are also consistent with the interpretation that non-optimal codons can cause ribosome stalling and premature termination *in vitro*. It should be noted that some premature terminated products could be seen for the OPT mRNA, indicating that translation premature termination can also occur independent of codon usage.

To determine the effect of codon usage on translation efficiency *in vivo*, we introduced the StopGo P2A peptide-containing eGFP-Luc reporter transgene into *Neurospora*. The P2A sequence triggers cleavage and release of nascent peptide during translation and allows the ribosome to continue translation of downstream mRNA sequence (81). This function of the 2A peptide has previously been analyzed in detail using *Neurospora* translation extracts (82). The 2A peptide was inserted between eGFP and different codon-optimized versions of *Luc*: *Luc* encoded by only optimal codons (2A-OPT), by optimized codons with the exception of WT codons in the middle of the protein (2A-M-WT), and by optimized codons in the N-terminal region and WT codons in the C-terminal region of the protein (2A-N-OPT) (Figure 4C). Because both eGFP and LUC are translated from the same mRNA, the effect of codon usage on translation efficiency of the luciferase mRNA can be determined by comparing the ratio of LUC signal to eGFP (encoding upstream of 2A). This approach enables accurate normalization because it can account for any effects of codon usage on mRNA levels; codon usage was previously shown to greatly affect *Luc* mRNA levels (12). These constructs were introduced at the *his-3* locus of a *dim-5^KO^ Neurospora* strain and eGFP and LUC levels were determined by western blot analysis using the Flag antibody. In the *dim-5^KO^* strain, the transcriptional effect mediated by rare codons in *Luc* is greatly reduced (12). The levels of eGFP-2A-LUC, LUC and eGFP were decreased in the 2A-M-WT strain and further decreased in the 2A-N-OPT strain compared to the 2A-OPT strain, consistent with the previously observed effect of codon usage on mRNA levels (Figure 4D and Supplementary Figure S3B). As expected, compared to the 2A-OPT strain, the ratio of LUC to eGFP signals was significantly lower in the 2A-M-WT strain and was reduced even further, by about 80%, in the 2A-N-OPT strain (Figure 4D and E). These results indicate that the use of non-optimal codons directly alters translation efficiency *in vivo* in *Neurospora*. However, we were not able to detect prematurely terminated LUC products in cell extracts, presumably because they were rapidly degraded in cells.

### Non-optimal codons cause ribosome stalling and premature termination in *Drosophila* cells

**Figure 5.**
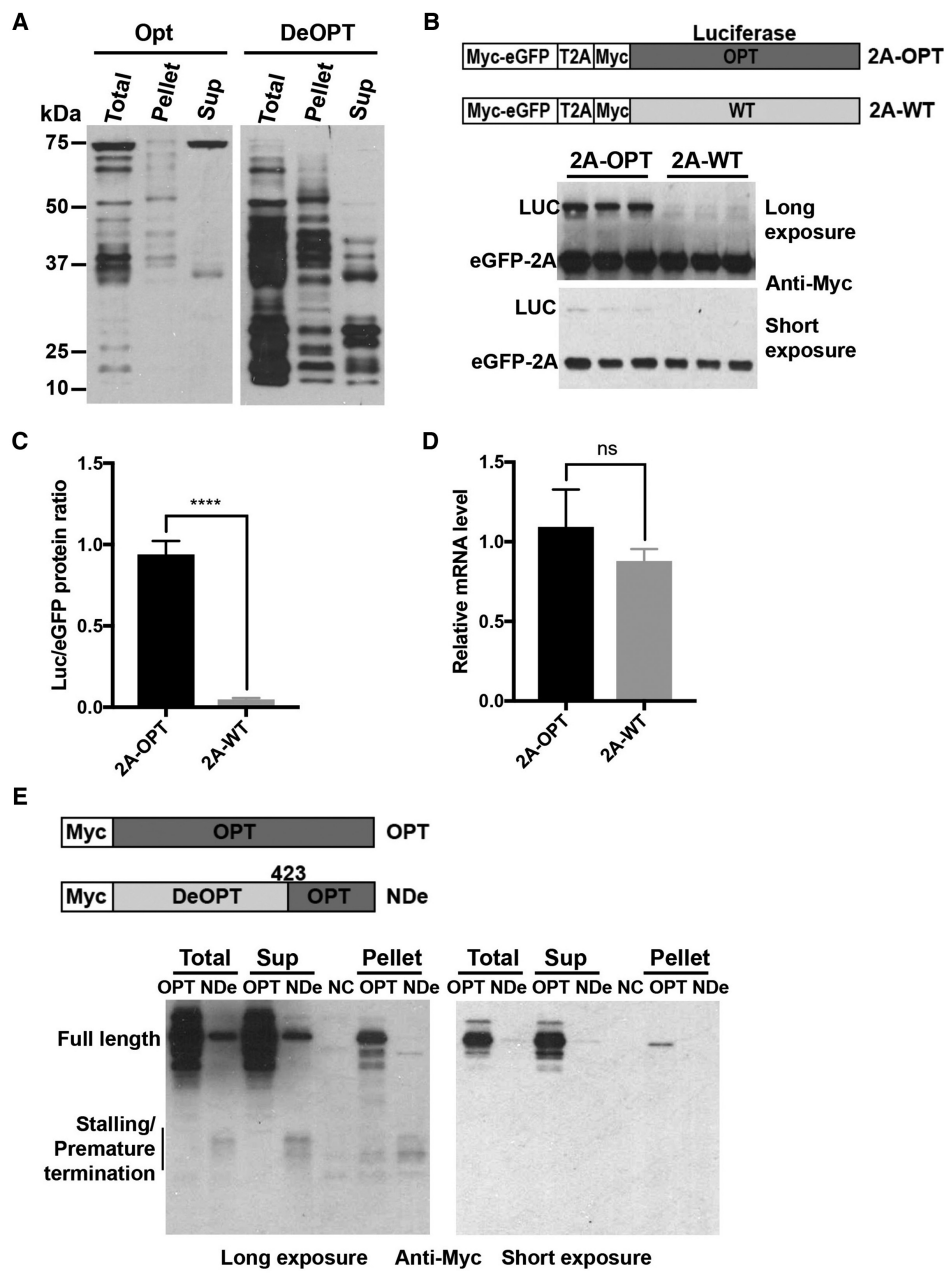
Non-optimal codons cause ribosome stalling and premature termination in *Drosophila* S2 cells. (**A**) Western blot analysis of total translation products of N-terminal c-Myc tagged *Luc* mRNAs and ribosome-associated nascent peptides using c-Myc antibody. OPT and DeOPT *Luc* mRNA were individually translated for 15 min before addition of cycloheximide (0.5 mg/ml final concentration). Ribosome-associated nascent peptides were separated by sucrose cushion centrifugation. Sup: supernatant. (**B**) Top panel: Diagrams showing the eGFP-2A-Luc reporter constructs used in S2 cell experiments. Bottom panel: Representative western blot analysis of products from eGFP-2A-Luc reporter constructs detected using c-Myc antibody. Two different exposures are shown. (**C**) Quantification of LUC/eGFP protein ratio from western blot analysis in panel (B) and two additional independent experiments. Data are means ± SD. ****P < 0.0001. (**D**) Relative mRNA levels from eGFP-2A-OPT and eGFP-2A-WT reporter constructs measured by RT-qPCR. Data are means ± SD. ns: not significant. (**E**) Top panel: Diagrams showing the *Luc* reporter constructs that were transfected individually into S2 cells. Bottom panels: Western blot analysis of total cell extracts and isolated ribosome-associated nascent peptides using c-Myc antibody; two exposures are shown. Sup: supernatant. Similar results were obtained from at least one replicate for experiments described in (A) and (E).

We next used a *Drosophila* cell-free translation assay to determine whether the effects of codon usage on translation are conserved in animal cells. Fully codon-optimized (OPT) or deoptimized (DeOPT) *Luc* mRNA was translated in an *in vitro* S2 cell extract prepared as previously described (29). After 15 min of reaction, some nascent luciferase peptides shorter than full-length were observed when the OPT *Luc* mRNA was translated, but the full-length LUC was the most prominent protein product (Figure 5A). In addition, the sucrose cushion centrifugation assay showed that almost all of the shorter products were present in the pellet (Figure 5A), indicating that they are peptidyl-tRNA intermediates associated with ribosome. In contrast, only a small amount of full-length LUC was produced from the DeOPT *Luc* mRNA; the vast majority of translation products were low molecular weight intermediates and many were found in the supernatant (Figure 5A), indicating that they are prematurely terminated products. In addition, more nascent peptide bands were observed in the DeOPT pellet than the Opt pellet, indicating there were more stalled peptidyl-tRNAs in the DeOPT. This result indicates that codon usage plays a major role in controlling translation efficiency *in vitro* and that rare codons result in ribosome stalling and premature termination in the animal system as well as in the fungal system.

To confirm the role of codon usage on translation efficiency *in vivo*, eGFP-2A-LUC fusion reporter constructs were created by inserting a T2A (similar to P2A) peptide between the regions encoding eGFP and LUC (Figure 5B). These constructs were examined using *Drosophila* S2 cells. Both eGFP and LUC are expressed with a 4xMyc tag at the N-termini. LUC was encoded by either optimal codons (2A-OPT) or WT codons (2A-WT). These constructs were individually transfected into S2 cells, and western blot was performed on extracts using a c-Myc antibody. Although the levels of the eGFP were comparable for both constructs, the levels of the Luc bands were dramatically different: LUC expression from the 2A-WT construct was almost undetectable (Figure 5B and C). qRT-PCR showed that these two constructs produced similar levels of mRNA (Figure 5D). These results demonstrate that codon usage has a major role in determining translation efficiency in S2 cells.

To further confirm that non-optimal codons can cause ribosome stalling and premature termination *in vivo*, we expressed Myc-tagged codon-optimized *Luc* (OPT) or *Luc* with N-terminal and middle region codons deoptimized (N-DeOPT) in S2 cells (Figure 5E). As expected, the level of full-length LUC was dramatically lower in the N-DeOPT cells (Figure 5E). In addition, some low molecular weight bands were only observed in the N-DeOPT cells, suggesting that they were stalled/premature terminated products.

To confirm the nature of these different Myc-tagged LUC protein species, we performed sucrose cushion centrifugation to separate the ribosome-associated nascent peptides and the terminated products. Similar molecular weight species were found both in the pellet and supernatant (Figure 5E), suggesting that non-optimal codons cause ribosome stalling and premature termination *in vivo*.

### eRF1 mediates premature termination of stalled ribosomes on rare sense codons

**Figure 6.**
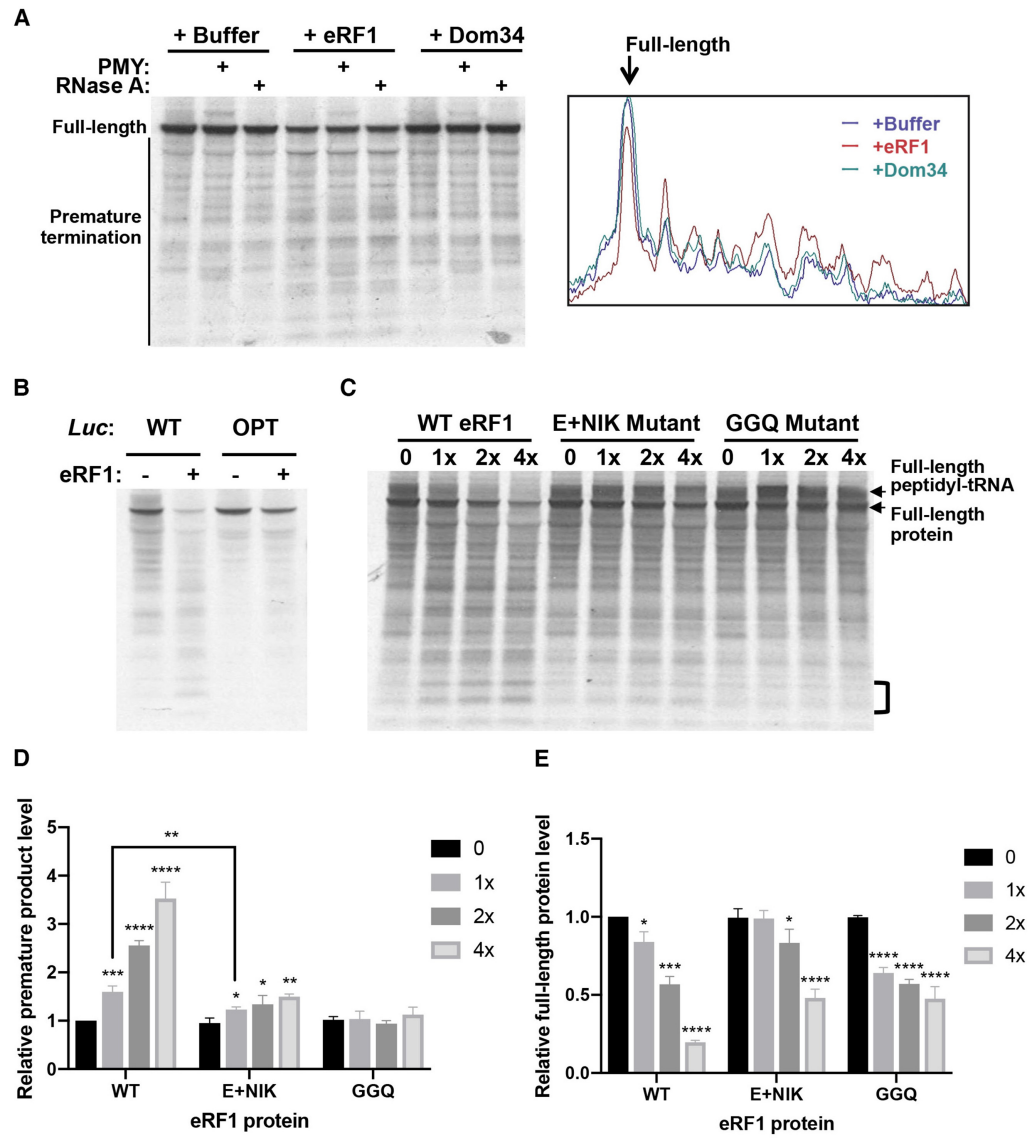
eRF1 results in premature termination at rare codons. (**A**) Left: [^35^S]-Met-labeled total translation products of the WT *Luc* mRNA with or without the addition of recombinant eRF1 or Dom34. RNase A (1 μg/μl) or puromycin (PMY, 1 μg/μl) was added after 15 min of translation. Right panel: ImageJ analysis of the translation products in the western blot lanes 1, 4 and 7. (**B**) [^35^S]-Met-labeled total translation products of the WT or OPT *Luc* mRNA with or without the addition of recombinant eRF1 (0.6 μg eRF1 protein in a 20-μl reaction). Similar results were obtained from at least one replicate for experiments described in (A) and (B). (**C**) [^35^S]-Met-labeled total translation products of the WT *Luc* mRNA with the addition of different concentrations of recombinant WT or mutant eRF1 protein (1× is 0.6 μg/20 μl reaction). (**D**) Quantification of premature termination based on bands with the region labeled by the bracket in panel (C) from three independent experiments. Data are means ± SD. *P < 0.05. **P < 0.01. ***P < 0.001. **** *P* < 0.0001. (**E**) Quantification of full-length LUC levels from independent experiments. Data are means ± SD. *P < 0.05. ***P < 0.001. **** *P* < 0.0001.

Stalled ribosomes are known to be rescued by the Dom34/Hbs1 complex in the quality control pathway, although recent studies also proposed an alternative mechanism mediated by the eRF1/eRF3 complex (50,51,83,84). To determine the mechanism for the premature translation termination mediated by rare codons, we expressed and purified recombinant *Neurospora* eRF1 and Dom34 using *E. coli*. *In vitro* translation reactions of the WT *Luc* mRNA in the *Neurospora* translation extract were performed in the presence of each of these proteins. After 6 min of reaction, harringtonine was added to inhibit translation initiation and the reaction was stopped after an additional 9 min. Using this protocol, the reaction will mainly reflect the elongation process. eRF1 but not Dom34 markedly increased the levels of the low molecular weight nascent peptide bands and decreased the levels of the full-length LUC (Figure 6A-B). Treatment with RNase A or puromycin demonstrated that most of these products were not peptidyl-tRNA species but were premature termination products. In addition, an increase of eRF1 concentration in the reactions led to a dose-dependent increase of premature terminated nascent peptides and decrease of the full-length LUC protein (Figure 6C, the first four lanes).

Comparison of *in vitro* translation products between the WT and OPT *Luc* mRNAs showed that the addition of eRF1 resulted in higher levels of the translation intermediates and a greater reduction of full-length luciferase for the WT mRNA than for the OPT mRNA (Figure 6B). This result demonstrates that effect of eRF1 on the production of translation intermediates and full-length protein is dependent on codon usage: eRF1 preferentially promotes premature termination at rare codons.

To further examine the role of the quality control pathway, we obtained the *Neurospora dom34* knock-out mutant and prepared translation extracts. *In vitro* translation assays using the WT and OPT luciferase mRNAs showed that the deletion of this critical component in the quality control pathway did not significantly affect the codon usage-dependent accumulation of the intermediate translation products (Supplementary Figure S4). Together, these results suggest that eRF1 but not Dom34 plays a major role in mediating premature translation termination when the ribosomes are stalled at rare codons in *Neurospora*.

eRF1 mediates normal translation termination due to its high specificity for the stop codons (59,61). To determine the role of the stop codon recognition domain of eRF1 on premature termination at rare sense codons, we generated a mutant eRF1 (E+NIK) in which the residues critical for stop codon recognition (E55 and NIK61–63) are mutated to alanines. In addition, we also generated the catalytically dead eRF1 (GGQ) mutant protein in which the residues required for hydrolysis of peptidyl-tRNA and nascent peptide release was mutated to alanines (62). Compared to the products produced in the presence of WT eRF1, the levels of prematurely terminated nascent peptides were reduced by the E+NIK eRF1 mutant protein (Figure 6C and D). In addition, the E+NIK eRF1 resulted in a marked increase of the a high molecular weight band corresponding to full-length LUC linked to tRNA and reduced the amount of full-length LUC compared to that observed in the presence of WT eRF1 (Figure 6C–E and Supplementary Figure S5B). These results indicate that the mutant eRF1 does not efficiently recognize the *Luc* stop codon. Because the E+NIK motif is involved in stop codon recognition, the fact that this mutant protein was still able to weakly promote the premature termination suggest that recognition of codons by eRF1 is not essential for premature termination. This interpretation is consistent with the role of eRF1 in causing premature termination at sense codons. In contrast, although the catalytically dead eRF1 (GGQ) protein resulted in an increase of full-length LUC linked to tRNA, it completely failed to increase premature termination products at sense codons (Figure 6C–E), indicating that premature termination at sense codons requires the eRF1 catalytic activity. Note that the decrease of the full-length peptide level for the GGQ samples were due to the failure to hydrolyze the flull-length peptidyl tRNA (Figure 6C and Supplementary Figure S5C).

**Figure 7.**
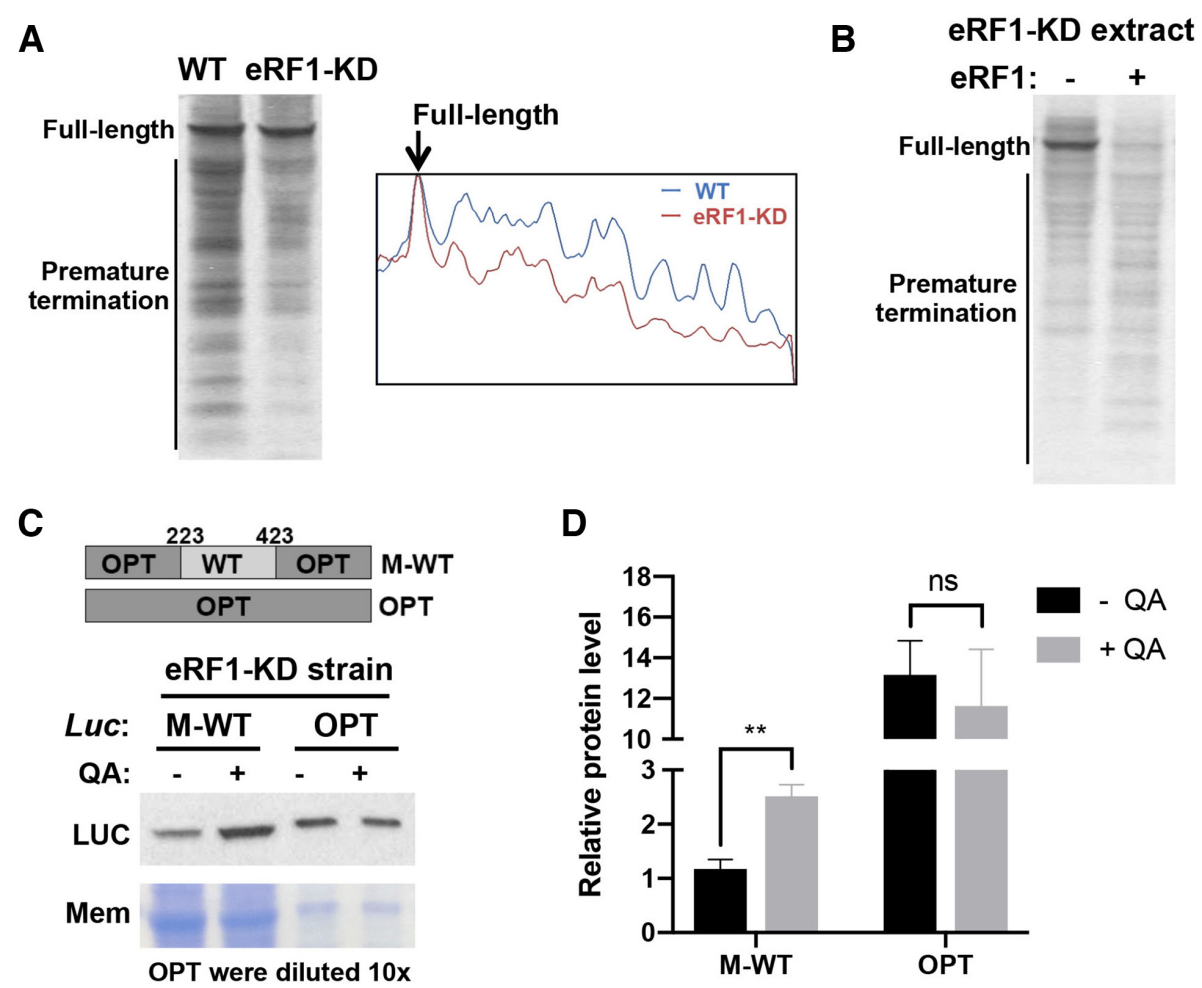
eRF1 regulates translation efficiency in *Neurospora*. (**A**) Left panel: [^35^S]-Met-labeled total translation products of WT *Luc* mRNA from experiments in cell-free translation extracts prepared from the WT or eRF1-depleted strain (eRF1-KD). Right panel: ImageJ analysis of the translation products. (**B**) [^35^S]-Met-labeled total translation products of the WT *Luc* mRNA with or without the addition of recombinant eRF1 in cell-free translation extracts prepared from the eRF1-depleted strain. (**C**) Top panel: Diagrams showing two luciferase-expressing constructs used for *Neurospora* transformation into the eRF1-KD strain. Bottom panel: Representative western blot analysis using luciferase antibody showing the levels of luciferase protein in the strains. cultureed with or without 1 mM QA. (**D**) Quantification of protein levels from western blot analysis in panel (C) and two additional independent experiments. Data are means ± SD. **P < 0.01. ns: not significant.

To further confirm the role of eRF1 in premature termination of sense codons, we generated a *Neurospora* strain (eRF1-KD) in which the expression of *eRF1* can be inducibly knocked-down (KD) by expression of a long double-stranded RNA in the presence of quinic acid (QA) (Supplementary Figure S6A) (85). Because eRF1 is an essential gene in *Neurospora*, inducible silencing allowed us to determine the function of eRF1 when its expression is temporarily silenced. We prepared translation extracts from the eRF1-KD strain grown in the presence of inducer and performed *in vitro* translation using the WT luciferase mRNA. As expected, the accumulation of the premature terminated nascent luciferase peptides was markedly reduced in the eRF1-KD extract compared to the lysate from the WT strain (Figure 7A). On the other hand, adding recombinant eRF1 protein into the eRF1-KD extracts markedly increased of premature terminated products and dramatically decreased the full-length luciferase level (Figure 7B), indicating that eRF1 directly promotes premature termination at rare sense codons. Moreover, by comparing the time of first appearance of LUC signal in the WT and eRF1-KD lysates (Supplementary Figure S6B), we confirmed that eRF1 does not appear to have a major effect on translation elongation rate (15). We also found the total translation level is low in the eRF1-KD lysate. This is because eRF1 is an essential gene and the silencing of its expression strongly impacted *Neurospora* global translation and growth rate.

To examine the role of eRF1 on codon usage-dependent translation efficiency *in vivo*, we introduced luciferase-expressing constructs (OPT and M-WT) with different codon usage profiles into the eRF1-KD strain and compared the effect of eRF1 knock-down on luciferase expression level. As shown in Figure 7C and D, the knock-down of eRF1 by the addition of QA resulted in a significant increase of the M-WT luciferase protein but did not affect the OPT luciferase level. On the other hand, the *Luc* mRNA levels were not significantly affected (Supplementary Figure S6C). These results suggest that eRF1 preferentially impairs translation efficiency of mRNA with poor codon usage *in vivo*. Together, our results here suggest that eRF1 regulates translation efficiency by competing with tRNAs for stalled ribosomes with empty A sites on rare sense codons to trigger premature translation termination.

## DISCUSSION

Codon usage is a major determinant of gene expression levels in both prokaryotes and eukaryotes (5–8,14,86). Although codon usage is proposed to regulate translation efficiency, the mechanisms by which it does this are unclear. Using *in vitro* translation assays and ribosome profiling, we previously showed that codon usage regulates the rate of translation elongation so that common codons are decoded faster during translation in both *Neurospora* and *Drosophila* cells (15,29). Here by using ribosome profiling, we evaluated ribosome occupancy for each individual codon in *Neurospora* and showed that for all of 18 amino acid codon families with two or more synonymous codons, the optimal codons were always decoded more rapidly than rare codons (Figure 1). By performing *in vitro* translation assays in a *Neurospora* cell-free system, we monitored the production of nascent peptides and showed that rare codons cause ribosome stalling through mechanisms that can be amino acid sequence context-independent and sequence context-dependent (Figures 2 and 3). In addition, such ribosome stalling can result in premature translation termination in *Neurospora* (Figure 4) and in *Drosophila* S2 cells (Figure 5), indicating that the effect of codon usage on translation efficiency is conserved in both fungi and animals.

Dom34 and eRF1 are both structural mimics of tRNA. The Dom34/Hbs1 complex has been shown to rescue stalled ribosomes through the no-go decay and non-stop decay pathways (49–51). Dom34 enters the A site of the ribosome to facilitate subunit dissociation, and Hbs1 promotes subunit dissociation (50,87). However, although Dom34 is related to eRF1, it lacks the eRF1 motifs for codon recognition and peptidyl tRNA hydrolysis. A recently reported high-resolution structural analysis of Dom34 and Hbs1 bound to a yeast ribosome with a non-stop mRNA revealed that a unique basic loop of Dom34 mimics mRNA at the A site, raising the possibility that Dom34 may recognize ribosome without mRNA at the A site (88). Although the Dom34/Hbs1 complex was previously proposed to rescue ribosome stalled at 3′ end of mRNA in the no-go decay pathway (49,50,54), it is not clear whether it is involved in premature termination at rare codons within open-reading frames. eRF1 is highly specific for stop codons because of extensive interactions between eRF1, the stop codon, 18S rRNA and ribosomal proteins (59,61). eRF1 enters ribosome A site and is responsible for stop codon recognition, whereas eRF3 acts to stimulate this process in a GTP-dependent manner (56–58). eRF1 induces hydrolysis of peptidyl-tRNA and the release of nascent peptide, stimulated by adenosine triphosphate-binding cassette protein ABCE1 (89). The highly specific recognition of stop codons by eRF1 at the A site prevents eRF1 from triggering termination at sense codons under normal conditions. Here, we showed that eRF1 but not Dom34 mediates the rare codon-dependent premature translation termination in *Neurospora* (Figures 6 and 7).

Our results suggest that eRF1 competes with tRNAs for recognizing sense codons and triggers termination. The mutation of the eRF1 NIKS motif critical for stop codon recognition reduced but did not abolish the ability of eRF1 to cause premature termination, suggesting that specific codon recognition is not required for termination (Figure 6C). This is consistent with the idea that, at rare codons, ribosomes are more likely to be stalled with an empty A site than at common codons, and therefore eRF1 would then be more likely to trigger premature termination at rare codons compared to common codons.

The demonstration that full-length polypeptide synthesis can be reduced by premature termination events at rare codons through eRF1 uncovers a mechanism by which codon usage could generally control protein synthesis efficiency. Consistent with our results, eRF1 has been previously shown to trigger termination at UGG, AGA, AAA and UAU codons. These codons are thought to structurally mimic stop codons and at ribosomes stalled in polylysine-coding regions (64–66,83). On the other hand, prokaryotic release factors have been shown to mediate peptide quality control after misdecoding in *E. coli* (90).

In addition to the role of codon usage in regulating translation efficiency, codon usage has also been shown to affect gene expression levels by influencing mRNA stability, transcription and transcriptional termination. The effect of codon usage on translation elongation rate can further regulate co-translational protein folding and translation fidelity. Therefore, codon usage represents a major regulatory ‘code’ that can control multiple aspects of gene and protein expression processes.

## Supplementary Material

gkz710_Supplemental_File
