# Supplementary material for: eRF1 mediates codon usage effects on mRNA translation efficiency through premature termination at rare codons
Source: Nucleic Acids Res. 2019 Aug 14;47(17):9243–58. doi: 10.1093/nar/gkz710 (PMC6755126; doi:10.1093/nar/gkz710)
Supplement: gkz710_Supplemental_File [file gkz710_supplemental_file.pdf]

Supplemental Figures

Figure S1. Quantification of [^{35}S]-Met-labeled protein bands of indicated Figures in Figure 2 and 3 by ImageJ.

Figure S2. (A) [^{35}S]-Met-labeled total translation products of WT *Luc* mRNA showing that the polypeptide species are stable after the addition of CHX. (B) Top panel: A diagram showing the stop-codon-less mRNA (StoplessNLUC), which contains N-terminus luciferase sequence (223 aa) with no stop codon followed by a 30 nt poly(A) tail. Bottom panel: [^{35}S]-Met-labeled total translation products of the *StoplessNLUC* mRNA. .After 10 min of translation, reactions were terminated by addition of cycloheximide or RNase A. Ribosome-associated nascent peptides were separated by sucrose cushion centrifugation. Sup: supernatant.

Figure S3. Expression level of eGFP-2A-LUC reporter constructs. (A) A long exposure of western blot result in Figure 4D. (B) Relative mRNA levels from eGFP-2A-OPT and eGFP-2A-WT reporter constructs measured by RT-qPCR. Data are means ± SD. *$P < 0.05$. **$P < 0.01$.

Figure S4. [^{35}S]-Met-labeled total translation products of WT or OPT *Luc* mRNA in cell-free translation extracts prepared from the wild-type and *dom34*^{KO} strains.

Figure S5. (A) [^{35}S]-Met-labeled in vitro translation products of the WT *Luc* mRNA using the WT *Neurospora* cell extracts in the presence of different concentrations of WT or mutant eRF1 proteins. 1x indicates 0.6 μM of eRF1. A short exposure of result in

Figure 6C is shown. (B) [^{35}S]-Met-labeled translation products of the WT *Luc* mRNA with the addition of GGQ mutant eRF1. RNase A (1 µg/µl) was added after 15 min of translation. (C) Quantification of the relative total levels of the full-length peptide and the full-length peptidyl-tRNA from independent experiments described in Figure 6C. Data are means ± SD. **P < 0.01. **** P < 0.0001. ns: not significant.

Figure S6. (A) RT-qPCR results showing the relative *eRF1* mRNA levels from the wild-type or eRF1-KD strains. Data are means ± SD. ***P < 0.001. (B) Real-time luciferase activity of WT *Luc* mRNA in cell-free translation extracts prepared from the wild-type or eRF1 knock-down strains. Recorded relative light units (RLUs) were plotted versus translation reaction time in 1-min intervals. Times of first appearance are indicated by arrows. Data are represented as mean ± SD. (C) Relative mRNA levels from OPT and M-WT reporter construct strains measured by RT-qPCR. Data are means ± SD. *ns: not significant.

Figure S1

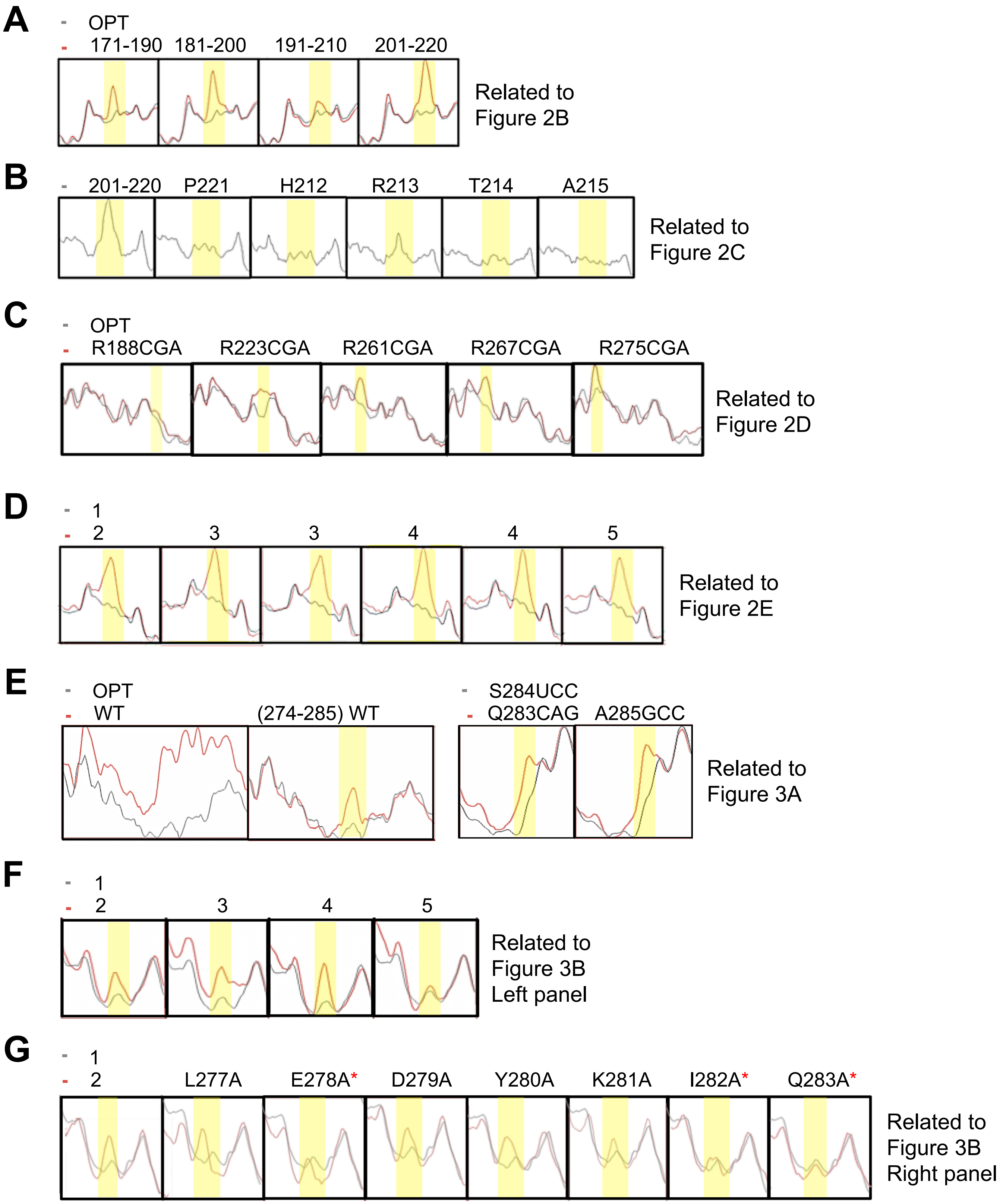

A
OPT
171-190
181-200
191-210
201-220
Related to Figure 2B
B
201-220
P221
H212
R213
T214
A215
Related to Figure 2C
C
OPT
R188CGA
R223CGA
R261CGA
R267CGA
R275CGA
Related to Figure 2D
D
1
2
3
3
4
4
5
Related to Figure 2E
E
OPT
WT
(274-285) WT
S284UCC
Q283CAG
A285GCC
Related to Figure 3A
F
1
2
3
4
5
Related to Figure 3B Left panel
G
1
2
L277A
E278A*
D279A
Y280A
K281A
I282A*
Q283A*
Related to Figure 3B Right panel

Figure S2

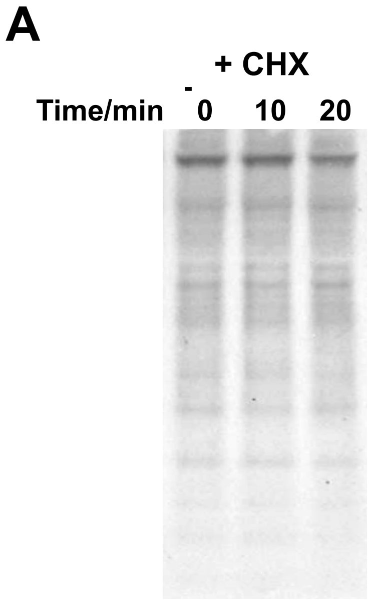
A
+ CHX
-
Time/min 0 10 20

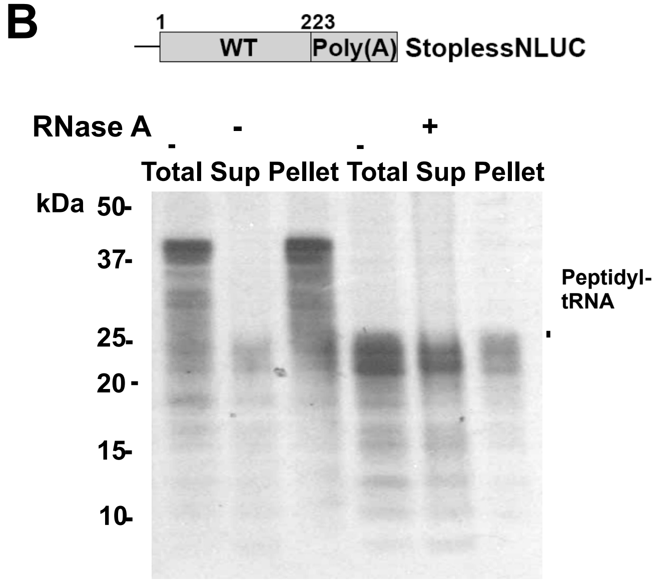
B
1
223
WT
Poly(A)
StoplessNLUC
RNase A - +
- -
Total Sup Pellet Total Sup Pellet
kDa 50-
37-
25-
20 -
15-
10-
Peptidyl-
tRNA

Figure S3

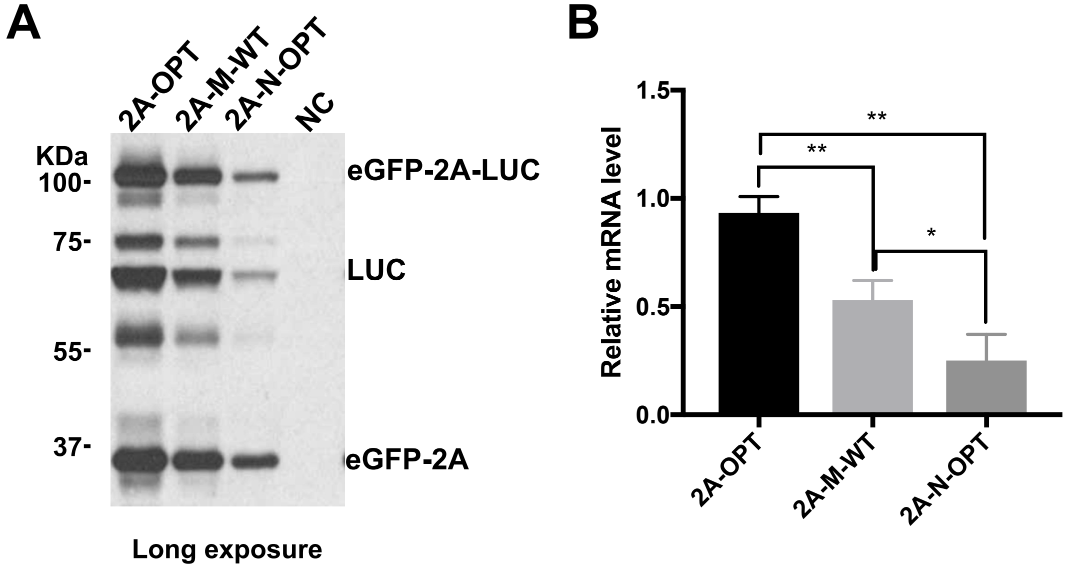
A
2A-OPT
2A-M-WT
2A-N-OPT
NC
KDa
100-
75-
55-
37-
eGFP-2A-LUC
LUC
eGFP-2A
Long exposure
B
1.5
1.0
0.5
0.0
Relative mRNA level
**
**
*
2A-OPT
2A-M-WT
2A-N-OPT

Figure S4

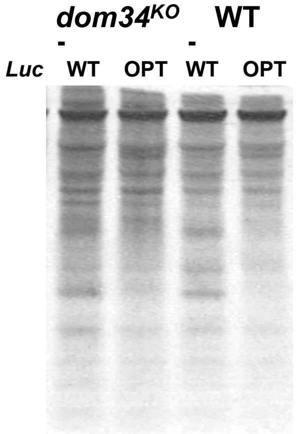

Figure S5

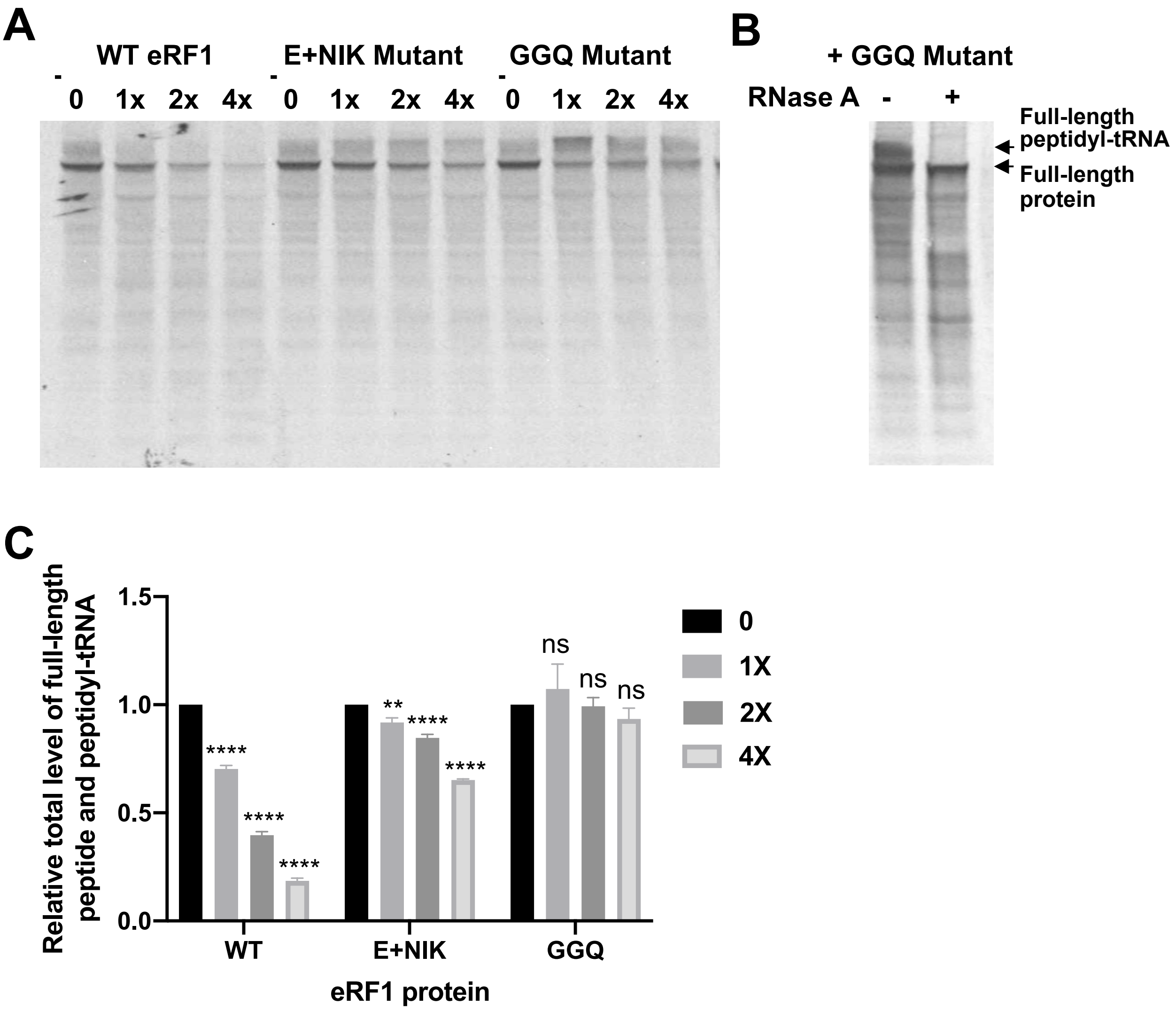
A
WT eRF1
E+NIK Mutant
GGQ Mutant
-
0
1x
2x
4x
B
+ GGQ Mutant
RNase A
-
+
Full-length peptidyl-tRNA
Full-length protein
C
Relative total level of full-length peptide and peptidyl-tRNA
1.5
1.0
0.5
0.0
****
**
ns
WT
E+NIK
GGQ
eRF1 protein
0
1X
2X
4X

Figure S6

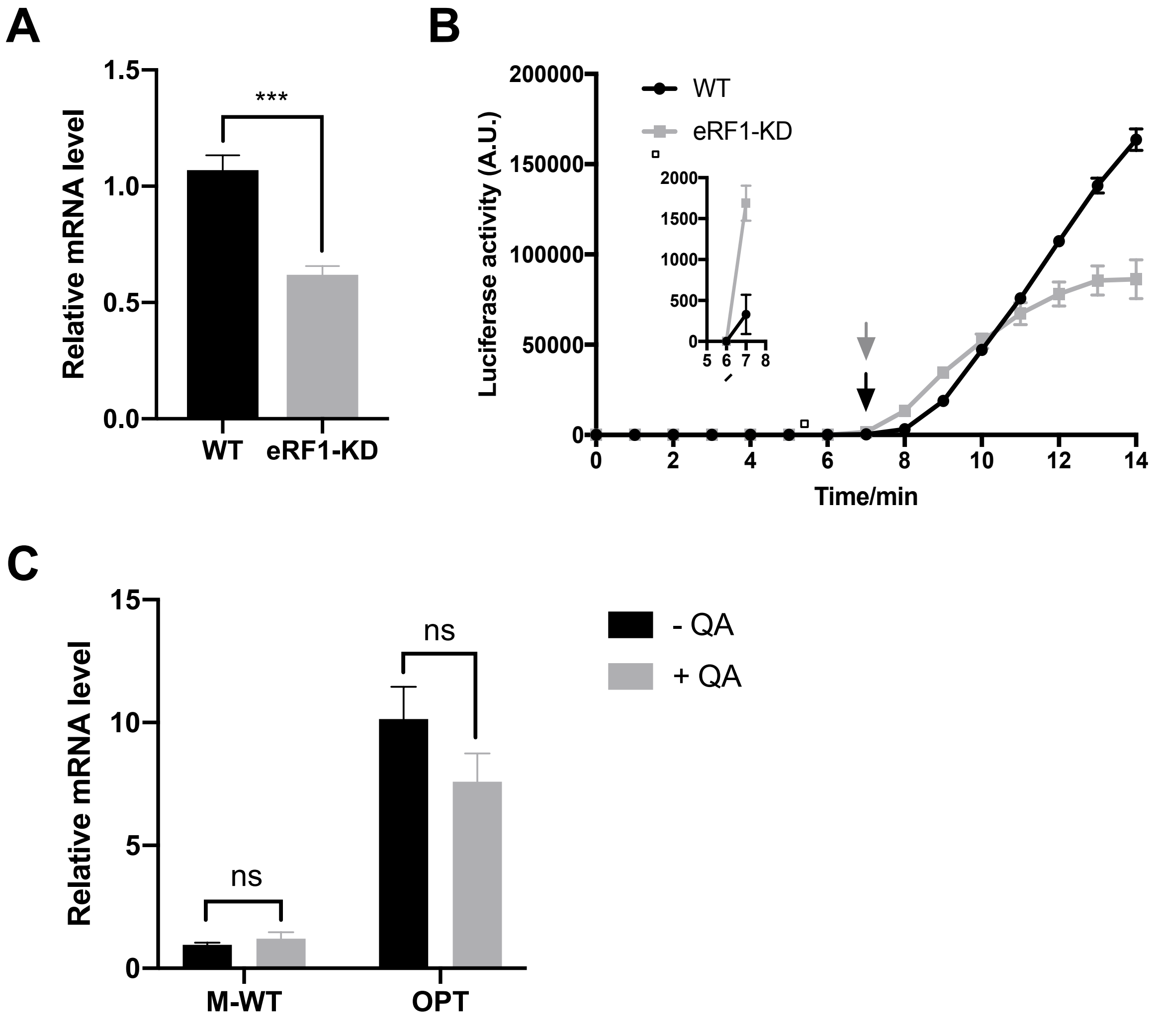

A
Relative mRNA level
1.5
1.0
0.5
0.0
***
WT
eRF1-KD
B
Luciferase activity (A.U.)
200000
150000
100000
50000
0
WT
eRF1-KD
2000
1500
1000
500
0
5
6
7
8
0
2
4
6
8
10
12
14
Time/min
C
Relative mRNA level
15
10
5
0
ns
ns
M-WT
OPT
- QA
+ QA
